# Repeatability and Agreement of Central Vault for Implantable Collamer Lens Obtained by the Tomey OA-2000 Biometer and Spectralis OCT

**DOI:** 10.1155/2024/3684626

**Published:** 2024-06-24

**Authors:** Hao Wu, Zuocheng Wang, Pengfei Wang, Yifei Meng, Zengying Wang, Yuhong Xue, Bohua Jiang, Shuaixi Pan, Zhipeng Yan

**Affiliations:** ^1^ Department of Ophthalmology Bengbu First People's Hospital, 229 Tushan Road, Bengbu 233000, China; ^2^ Department of Ophthalmology The Third Hospital of Hebei Medical University, 139 Ziqiang Road, Shijiazhuang 050000, China; ^3^ School of Management Shijiazhuang Tiedao University, Shijiazhuang 050043, China

## Abstract

**Objective:**

To assess repeatability and agreement of central vault for implantable collamer lens (ICL) measured by the Tomey OA-2000 biometry and Spectralis optical coherence tomography (OCT).

**Methods:**

In this prospective study, the central vault was measured by the Tomey OA-2000 biometer and Spectralis OCT in 84 eyes (43 patients) after ICL implantation at six month follow-up. Three consecutive scans were obtained by one experienced technician using Tomey OA-2000 and the Spectralis OCT in the same day. The coefficient of variation (CoV), intraclass correlation coefficient (ICC), within-subject standard deviation (Sw), and 2.77 Sw were calculated to assess the repeatability and reproducibility. The paired *t*-test and Bland–Altman plots were used to analyze the differences and agreements of central vault measured by two devices.

**Results:**

Repeatability of the central vault measured by Tomey OA-2000 biometer and Spectralis OCT showed that the CoV was 2.71% and 1.66%, respectively. The ICC for both devices was 0.996 and 0.999, respectively. The paired *t*-test showed that central vault measured by Tomey OA-2000 biometer was −7.25 ± 23.57 microns lower than that measured by Spectralis OCT (*P* = 0.006). The mean difference between measurements for Tomey OA-2000 and ASM-OCT with 95% limits of agreement (LoAs) was −38.94 to 53.44 *μ*m.

**Conclusion:**

Both Tomey OA-2000 biometer and Spectralis OCT displayed good repeatability for the measurement of central vault of ICL. Good reliability and agreement were observed between Tomey OA-2000 biometer and Spectralis OCT. Both instruments are useful but not replaced each other for central vault measurements.

## 1. Introduction

Myopia is one of the most common eye diseases in the world [1]. There are more than 2 billion myopic individuals, which include 277 million high myopia patients [2]. Implantable collamer lens (ICL) implantation could correct a wider range of myopia and became an important surgical option for myopia and especially for high myopia patients [3].

Many studies reported that ICL implantation was a safe, effective, and even reversible surgical approach for myopia correction. Appropriate ICL central vault is considered to be highly related to the success and safety of ICL implantation. Generally, a desirable central vault from the central posterior surface of the ICL to the anterior surface of the crystalline lens is about 250–750 *μ*m for V4 model [4]. Extra high vault is a potential risk for angle closure and high intraocular pressure, whereas extra low vault could be the reason of the anterior subcapsular cataract (ASC) or cataract. However, in terms of V4c ICL with a central hole, Gonzalez-Lopez et al. found a good long-term tolerance of the crystalline lens and protection against anterior subcapsular cataract to low vaulting compared with V4 ICL without a central hole [5]. The precise measurement of central vault is highly emphasized. Several anterior segment imaging instruments have been developed to objectively visualize and evaluate central vault. Optical coherence tomography (OCT), Pentacam analysis system, and ultrasound biomicroscopy (UBM) are now widely used [6–8].

Tomey OA-2000 biometer is a device based on optical low coherence reflectometry (OLCR) which is now commercially available for ocular biometry. The anterior segment parameters could be automatically measured without the need of realignment [9, 10]. Several studies have showed excellent repeatability and reproducibility of Tomey OA-2000 for anterior segment ocular parameter measurements [9–11]. However, Tomey OA-2000 has not been applied for central vault measurement although it is theoretically possible. Spectralis OCT, combined with the anterior segment module (ASM) added on lens and accompanied by a software package build in it, is also offers high resolution cornea, sclera and anterior chamber angle images [12]. However, to the best of our knowledge, it has not been tried for central vault measurement yet. The purpose of this study was to evaluate agreement and repeatability of central vault obtained by the Tomey OA-2000 biometer and ASM-OCT.

## 2. Subjects and Methods

All subjects enrolled in this study underwent V4c (EVO Visian ICL) model implantation for correction of moderate to high myopia with or without astigmatism between September 2019 and March 2022 in the department of ophthalmology of the Third Hospital of Hebei Medical University. The point to emphasize is 8 patients younger than 21 years old were involved in this study. All these 8 patients signed the extra content for ICL implanting beyond indications, whom thoroughly understand the complication and accepted this procedure of own volition. ICL implantation operations were performed by the same experienced surgeon (Z.P.Y) according to the regular procedures [8]. The implantable collamer lens power calculations were completed by STAAR Surgical Company using a modified formula. The sizes of lens were selected depending on the corneal horizontal white-to-white value and anterior chamber depth measured by Pentacam analysis system (Oculus, Wetzlar, Germany). The inclusion criteria were patients with central vault between 100 *μ*m and 1000 *μ*m (measured by ASM-OCT) and no surgery-related complication after ICL surgery. All patients were well evaluated during routine follow-up examination. This study followed the Declaration of Helsinki and was approved by Ethical Committee Review Board of the Third Hospital of Hebei Medical University. All subjects provided written informed consent when the purpose of the study was explained to them in detail.

Sample-size calculation was performed a priori with the F-test ANOVA repeated measures method by Gpower 3.1 software. The parameter settings are as follows: Number of groups = 2, Number of measurements = 3, Corr among rep measures = 0.5, and Effect size *f* = 0.255. Using a 2-sided level of significance *α* = 0.05 and Power of 1-*β* = 0.8, the sample-size calculation indicated that a minimum of 84 samples would be required.

### 2.1. Central Vault Measurement by Tomey OA-2000 and ASM-OCT

**Figure 1:**
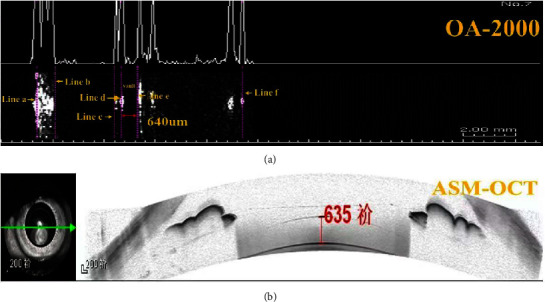
Vault measurements made using the OA-2000 biometer (a) between the anterior surface of the crystalline lens (line d) and the posterior surface of the ICL (line e); line a: anterior surface of the cornea; line b: the posterior surface of the cornea; line c: the anterior surface of the ICL; line d: the posterior surface of the ICL; line e: the anterior surface of the crystalline lens; line f: the posterior surface of the crystalline lens. Central vault measurements made using the ASM-OCT (b). Vault was measured between the anterior surface of the crystalline lens and the posterior surface of the ICL.

A single experienced operator performed 3 consecutive scans at 6 months after ICL implantation with both Tomey OA-2000 biometer (Tomey, Nagoya, Japan) and ASM-OCT (Heidelberg Engineering, Heidelberg, Germany), respectively. To control the bias between 3 consecutive vault measurements, all subjects accept training before examination so as to be well cooperated with good fixation ability when measurement. All measurements were operated in the morning and under the same dim light condition to minimize variations in the results. Patients sat in front of the devices, put their chin on the chinrest, focused on the target accordingly, and opened their eyes wide after blinking before each scan. The whole procedure was completed within 15 minutes, and only qualified measurements were adopted. In terms of Tomey OA-2000 biometry, a central averaged depth profile (A-scan) is presented in Figure 1(a). The averaged A-scan shows light intensity reflected from different structures of the eye. The first two peaks arise from the anterior and posterior surfaces of the cornea (line a and line b). Next two peaks arise from anterior and posterior surface of ICL (line c and line d) and last two peaks arise fromanterior capsule and posterior surface of crystalline lens (line e and line f) (as shown in Figure 1(a)). In detail, line a, line d, and line f were automatically displayed at the anterior surface of cornea, the posterior surface of ICL, and the posterior surface of crystalline lens, respectively. Line e was manually made at the anterior surface of crystalline lens. Then the central vault was defined as the distance between line a and line e minus the distance between line a and line d. It is necessary to emphasize that line e should be set up at the correct position. The precision of the caliper tool of the Tomey OA-2000 is 10 microns. For ASM-OCT, the vault was defined as the central distance from the back surface of the ICL to the anterior surface of the lens capsule (as shown in Figure 1(b)). The details of measurements by ASM-OCT were similar to those of anterior segment OCT reported from other previous studies [11, 13].

### 2.2. Statistical Analysis

All data were analyzed using SPSS software for Windows version 25 (IBM corporation, USA) and MedCalc statistical software (version 20.027, MedCalc Software Inc, Belgium). The sample size was analyzed and decided by Gpower 3.1 software. The distribution of all the datasets was analyzed for normality using Kolmogorov–Smirnov tests (*P* > 0.05). To determine the repeatability for two instruments, intraclass correlation coefficient (ICC) with absolute agreement, within-subject standard deviation (Sw), and within-subject coefficient of variation (CoV) was calculated for 3 consecutive measurements. The test-retest repeatability was defined as 2.77Sw, which indicated the interval within which 95% of the differences between measurements are expected to lie. The CoV was calculated as the ratio of the Sw to the overall mean. A smaller CoV means that the repeatability was higher. The ICC (ranging from 0 to 1) assesses the consistency for datasets of repeated measurements. The closer the ICC is to 1, the better the measurement consistency is, and a value more than 0.9 indicates acceptable clinical reliability. To compare central vault obtained by 2 devices, a paired *t*-test was applied to identify pairs that had significant differences. Bland–Altman plots were constructed to assess the repeatability of measurements with every device and agreement of measurements between 2 devices. The 95% limits of agreement (LoAs) were defined as ±1.96 standard deviation. A narrower 95% LoA indicated better agreement between measurements. All tests were two tailed analyzed, with *P* values less than 0.05 considered statistically significant.

## 3. Results

This study included 84 eyes of 43 patients (7 males and 36 females) that underwent ICL implantation. The mean age was 27.72 ± 6.94 years (range, 18 to 48 years old). The preoperative spherical equivalent (SE) was −8.60 ± 3.05D. Implanted ICL power was −9.79 ± 3.12D (Sphere, *n* = 84) with 1.43 ± 0.78D (Astigmatism, *n* = 41). Preoperative intraocular pressure (IOP) was 15.48 ± 2.59 mmHg. No complication occurred or was observed during the surgery and follow-up time. The details of all subjects' characteristics and ICL information are shown in Table 1.

### 3.1. The Repeatability of Central Vault Measured by Tomey OA-2000 Biometer and ASM-OCT

Table 2 shows the repeatability of the vault measured by Tomey OA-2000 biometer and ASM-OCT. Both CoV values were lower than 1.0% with 2.71% and 1.66%, respectively. Both ICC values displayed 0.996 and 0.999, respectively. The repeatability values (2.77 Sw) for both devices were 28.016 and 16.775 microns. In the repeated measures analysis of variance, *P* values were greater than 0.05 (0.649 and 0.998) which indicated that there was no significant difference in the repeated measures using the same instrument. This result showed high repeatability of vault values measured by Tomey OA-2000 or ASM-OCT.

### 3.2. Reliability and Agreement between Tomey OA-2000 Biometer and ASM-OCT

**Figure 2:**
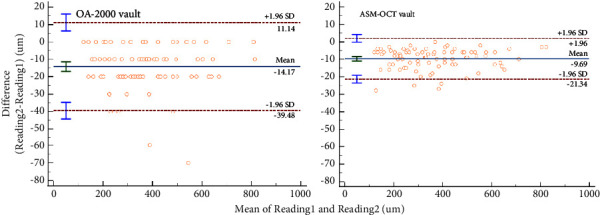
Bland–Altman plots displayed repeatability for three vault measurements by OA-2000 and ASM-OCT, respectively. Reading 1 = minimum of three measurements of vault; reading 2 = maximum of three measurements of vault. Reading 2 − reading 1 = range. The vertical axis represents the difference between these measurements and the horizontal axis shows the corresponding mean value. The 95% LoA is indicated using dashed lines, and the middle bold line represents the mean difference between these measurements.

**Figure 3:**
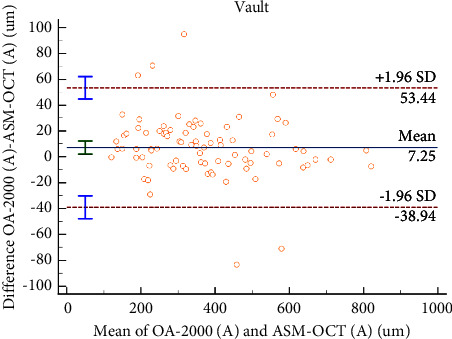
Bland–Altman plots comparing the level of agreement between OA-2000 and ASM-OCT measurements of vault. The vertical axis represents the difference between these measurements and the horizontal axis shows the corresponding mean value. The 95% LoA is indicated using dashed lines, and the middle bold line represents the mean difference between these measurements; A = average of three vault measurements.

The average of 3 measurements of vault by the Tomey OA-2000 was used to compare with that measured by ASM-OCT. The paired *t*-test showed that the vault value measured by Tomey OA-2000 was significantly higher than that measured by ASM-OCT (*P*=0.006) (Table 3). Repeatability of three central vault measurements for both Tomey OA-2000 and ASM-OCT was displayed using Bland–Altman plots (Figure 2). Agreement of central vault measurements between two methods was illustrated using Bland–Altman plots (Figure 3). The mean difference between measurements of Tomey OA-2000 and ASM-OCT with 95% limits of agreement (LoAs) was −38.94 *μ*m to 53.44 *μ*m. This result indicates that repeatability and agreements of the central vault measured by two devices were relatively good.

## 4. Discussion

One of the important parameters to be assessed after ICL implantation was the central vault [14]. There are several devices that have been used to measure central vault after ICL implantation, such as anterior segment optical coherence tomography (AS-OCT), Pentacam, and ultrasound biomicroscopy (UBM) [15]. All these devices have been proved for well precision (repeatability and agreement) in many previous studies [7, 8].

The Tomey OA-2000 biometer, which based on the principles of SS-OCT (Swept-Source Optical Coherence Tomography) and Placido disk topography, was designed for measuring axial length and anterior segment parameters including central corneal thickness (CCT), ACD, and lens thickness [16]. Although Tomey OA-2000 biometer could not calculated central vault values automatically like as other anterior segment parameters, A-scan profile images achieved by Tomey OA-2000 clearly displayed six peaks arised from anterior and posterior surface of the cornea, ICL and the crystalline lens. As a result, it could be used for central vault masurement. The central vault between peak line d (the posterior surface of ICL) and peak line e (anterior capsule of crystalline lens) was measured manually by one experienced technician. That the ICC value(>0.98) for central vault measured by Tomey OA-2000 displayed highly repeatability, which was comparable with other ocular parameters measured by this instument (ICC = 0.91–1.00) [8]. As a result, it should be one choice for central vault evaluation in clinics. Subsequently, we choose ASM-OCT for central vault measurement and compared it with Tomey OA-2000 for agreement examination.

The Spectralis anterior segment module (ASM) is an add-on lens, accompanied by a software package that can be added to the Spectralis SD-OCT (spectral domain OCT) device. ASM-OCT was different from anterior segment OCT (AS-OCT) which is being routinely accepted as the method of choice for central vault measurements [17]. The SS-OCT technology with longer wavelength light sources (1300 nm) delivers high-resolution images of the anterior segment along with a large image depth, at a fast acquisition speed, but ASM-OCT is SD-OCT using shorter wavelength light sources (820–880 nm), which is allowed for anterior segment imaging by special module. Although it cannot show entire anterior segment in one image like AS-OCT displayed, the ASM-OCT can offer similarly high-resolution images of the cornea, sclera, and anterior chamber angle, respectively [18]. Intriguingly, to the best of our knowledge, there is no study reported for central vault measurement with ASM-OCT. In fact, it can display the perfect ICL contour in the anterior segment of the eye after implantation. The current study proved that the pretty high repeatability (ICC more than 0.99) for central vault measured by ASM-OCT, but images capture need longer study curve for technician. Although lack of comparison data between ASM-OCT and AS-OCT, Similar scanning images suggested that ASM-OCT was comparable with the classical AS-OCT in terms of repeatability of central vault measurement [8]. In this study, the repeatability values (2.77 Sw) for both devices were 28.016 and 16.775 microns. Because a desirable vault height is between 250 and 750 microns, the different values between 2 consecutive vault measurements are clinically meaningful, especially in patients with low vault values. For that matter, both AS-OCT and Tomey OA-2000 may not be fully competent for postoperatively evaluating the safety of myopia patients after ICL implantation, especially for measuring low vault.

To the best of our knowledge, there is no study to investigate the precision of central vault measured by two devices. This study first evaluated the precision of central vault measured by Tomey OA-2000 and ASM-OCT and found excellent repeatability. In terms of LoAs values and *P* values, two devices displayed poor agreement and could not be used interchangeably for evaluating the central vault after ICL implantation. Central vault values with OA-2000 were significantly lower than that with ASM-OCT by a mean of 7.25 microns. In fact, it is not uncommon that there are statistically significant for anterior segment parameters measured by different devices [7, 8]. Wan et al. reported that AS-OCT showed higher central vault values than that of Pentacam system and UBM, while Pentacam showed lower measurements than that of UBM [8]. Several reasons might explain the difference among two instruments in current study. Firstly, Tomey OA-2000 and ASM-OCT have different resolution and lead to the different measured values. Secondly, the difference of scan location may contribute to the disagreement among instruments. The central vault also cauld be dynamic changed with pupil movement under different background illumination. Besides, the different refractive indices between ICL material and aqueous humor would have effect on the measurement of all structures lying behind the ICL. In terms of the axial revolution, OA-2000 and Spectralis OCT was 10 microns and 7 microns respectively [13], so a 6-micrometer difference with a LoA of below 50 microns seems a small difference for both devices [19]. That result demonstrated the vault values measured by two devices are pretty close although they cannot replace each other for central vault measurements. In brief, both ASM-OCT and Tomey OA-2000 could provide objective ways for central vault measurements after ICL implantation, but that could not determine which one was more accurate as lacking of a gold standard measurement so far

There are some limitations in this study. First, this study only focused on Chinese eyes, sample size was small, and more subjects are needed for further examination. Second, this study only explored Tomey OA-2000 and ASM-OCT for central vault measurements; other popular instruments including AS-OCT and UBM were not involved for comparison. Further research on the consistency of multiple instruments is required. Due to the changes in pupil diameter depending on different light conditions could induce the dynamic variations in vault, [19–21] the measurement of the vault was not in the same light condition is another limitation of this study. Additionally, a learning curve is necessary becaused central vault is manully measured on A-scan profile by Tomey OA-2000. There is also no specific build-in modulation available for central vault measurement for both devices.

## 5. Conclusions

This study reported the novel application of Tomey OA-2000 and ASM-OCT for central vault measurement after ICL implantation. Both devices displayed highly repeatable for central vault measurement. However, two devices displayed poor agreement and could not be used interchangeably for evaluating the central vault after ICL implantation.

## Figures and Tables

**Table 1:** Patients and study eye characteristics (*n* = 43 patients).

Demographic characteristics (*n* = 84 eyes)	Mean ± SD	Range
Age (years)	27.72 ± 6.94	18 to 48

*Gender*		
Female	36	83.72%
Male	7	16.28%

SE (D)	−8.60 ± 3.05	−2.75 to −15.88

ACD (mm)	3.24 ± 0.21	2.89 to 3.85

IOP (mmHg)	15.48 ± 2.59	9.35 to 20.18

WTW (mm)	11.29 ± 0.46	9.87 to 12.31

Size (mm)	12.1 mm (*n* = 27)	32.14%
	12.6 mm (*n* = 47)	55.95%
	13.2 mm (*n* = 10)	11.90%

Lens power (D)	Sphere: −9.79 ± 3.12 (*n* = 84)	−3.50 to −16.50
	Astigmatism: 1.43 ± 0.78 (*n* = 41)	0.50 to 4.00

Vault (microns)	OA (a) 371.55 ± 158.49	120 to 820
	OA (b) 372.02 ± 158.22	120 to 820
	OA (c) 372.98 ± 158.32	120 to 810
	OCT (a) 364.20 ± 163.13	117 to 822
	OCT (b) 365.65 ± 163.73	113 to 825
	OCT (c) 364.95 ± 163.44	123 to 825

**Table 2:** Repeatability of OA-2000 and ASM-OCT in measuring vault (*n* = 84).

Parameter	Mean vault	CoV (%)	Sw	2.77 Sw	*F*	*P* value	ICC (95%)
OA-2000	372.18	2.71	10.114	28.016	0.433	0.649	0.996 (0.994–0.997)
ASM-OCT	364.94	1.66	6.056	16.775	0.002	0.998	0.999 (0.999–1.000)

OA-2000: OA-2000 biometer; ASM-OCT: anterior segment module of Spectralis OCT; ICC: intraclass correlation coefficient; vault: distance between the back surface of ICL and the front surface of the lens. *P* values less than 0.05 were considered statistically significant.

**Table 3:** Comparison of vault measurements using OA-2000 and ASM-OCT (*n* = 84).

Parameter	Mean difference ± SD	95% CI	LoA	*t* value	*P* value
OA-2000 vs ASM-OCT	−7.25 ± 23.57	−12.36–−2.13	−38.94 to 53.44	−2.818	0.006^*∗*^

Paired samples test was used to compare measurements. *P* values less than 0.05 were considered statistically significant. Asterisk means the vault value was significantly different between OA-2000 and ASM-OCT.

## Data Availability

All the supporting data are included within the article.
